# Combinatorial inhibition of IGF2BP3-regulated receptor tyrosine kinases offers a new therapeutic strategy for triple-negative breast cancer

**DOI:** 10.1186/s13046-026-03686-7

**Published:** 2026-03-11

**Authors:** Zi-Wen Wang, Xu Zhang, Yu-Xin Wang, Meng-Yuan Cai, Jiao Chen, Ming-Yi Sang, Hai-Yan Yang, Liang Shi, Ji-Fu Wei, Qiang Ding

**Affiliations:** 1 Jiangsu Breast Disease Center, Department of General Surgery, The First Affiliated Hospital with Nanjing Medical University, Nanjing, 210029 China; 2 Department of Pharmacy, Jiangsu Cancer Hospital & Jiangsu Institute of Cancer Research & The Affiliated Cancer Hospital of Nanjing Medical University, Nanjing, 211166 China; 3 Affiliated Hospital of Integrated Traditional Chinese and Western Medicine, Nanjing University of Chinese Medicine, Jiangsu Province Academy of Traditional Chinese Medicine, Nanjing, China

**Keywords:** Receptor tyrosine kinase, IGF2BP3, N6-methyladenosine, Cynaroside, TNBC

## Abstract

**Background:**

Epidermal growth factor receptor (EGFR), characterized by its high positivity rate and strong specificity in triple-negative breast cancer (TNBC), has long been recognized as one of the characteristics of this subtype. However, EGFR inhibitors have demonstrated limited clinical efficacy in TNBC treatments. Previous studies revealed that multiple receptor tyrosine kinases (RTKs) were activated in TNBC. These RTKs cooperate through dimerization to activate downstream pathway phosphorylation, rendering EGFR monotherapy ineffective. This highlights the necessity for combinatorial strategies targeting multiple RTKs to treat TNBC.

**Methods:**

Published bulk-RNA sequencing datasets from erlotinib resistant TNBC patient-derived xenografts model revealed changes in gene sets in erlotinib resistant tissues. RNA immunoprecipitation and methylated RNA immunoprecipitation sequencing were used to detect co-regulated RTKs by insulin-like growth factor 2 mRNA-binding protein 3 (IGF2BP3). RNA pull-down and molecular docking clarified the interaction sites between IGF2BP3 and RTKs. Computational virtual docking, hierarchical screening, microscale thermophoresis and cytotoxicity assays were employed to screen small-molecule inhibitors aimed at disrupting IGF2BP3-RTK interactions. The determination of drug synergistic effects was achieved through the calculation of the combination index.

**Results:**

In this study, we identified IGF2BP3 could coordinately regulate multiple RTKs’ mRNA through an m6A-dependent manner and activate RTKs expression, and thus could be the key point to address the drug resistance problem. After clarifying the interaction sites between IGF2BP3 protein and RTK mRNAs, we identified cynaroside, a small-molecule inhibitor capable of disrupting IGF2BP3-RTK interactions, as a promising enhancer of tyrosine kinase inhibitor (TKI) efficacy. Moreover, we proposed a novel therapeutic strategy by combining cynaroside with TKI and chose the EGFR/MET bispecific antibody amivantamab for the best candidate in TNBC target treatment.

**Conclusions:**

Our study provide a mechanistic insight into IGF2BP3-mediated EGFR inhibitor resistance and present an emerging combinatorial approach to overcome therapeutic limitations in TNBC.

**Supplementary Information:**

The online version contains supplementary material available at 10.1186/s13046-026-03686-7.

## Introduction

Triple-negative breast cancer(TNBC) is the most aggressive subtype of breast cancer. Its formation is attributed to the lack of targeted therapeutic receptors- estrogen (ER), progesterone (PR), and human epidermal growth receptor-2 (HER2) [1]. This categorization gives TNBC a high degree of heterogeneity and also poses challenges for its targeted therapy [2]. Fortunately, large-scale genome sequencing reveals specific gene expression patterns and uncovers promising therapeutic targets in TNBC [3]. Among them, epidermal growth factor receptor༈EGFR༉has a unique amplification in TNBC (accounting for about 60%) and is long been recognized as one of the features of TNBC [3]. EGFR-targeted therapies have achieved remarkable success in the treatment of many cancers with EGFR overexpression [4–6]. For example, monoclonal antibodies against EGFR (e.g. cetuximab, panitumumab) combined with chemotherapy to treat RAS/BRAF wild-type metastatic colorectal cancer has been incorporated into the Chinese society of clinical oncology guideline [4]; cetuximab has been approved as a radiosensitizer for locally advanced head and neck squamous cell carcinoma (HNSCC) [5]. However, multiple clinical trials of the treatments of EGFR inhibitors in TNBC have ended in failure [7]. Cetuximab in combination with carboplatin produced responses in fewer than 20% of stage IV TNBC patients [8]. It suggests that there may exist specific mechanisms in their resistance to EGFR inhibitors in TNBC and need to be explored and overcame.

EGFR belongs to the ErbB family of receptor tyrosine kinases (RTKs), with other members of the family are HER2, HER3 and HER4 [9]. Previous study demonstrated that the activation of multiple RTKs was prevalent in TNBC [10, 11]. When EGFR being inhibited, other RTKs could still mediate the phosphorylation of signaling pathways causing ineffectiveness of EGFR inhibitors in TNBC [12]. Moreover, different from colorectal cancer and HNSCC, EGFR overexpression in TNBC mainly results from post-transcriptional modifications instead of genomic amplification (2%) [13, 14]. This flexible regulation allows compensatory activation of multiple RTKs and promotes resistance to EGFR inhibitors [15]. Therefore, to develop effective targeted therapies for TNBC, it is necessary to investigate the strategies involving post-transcriptional modifications for the coordinated regulation of RTKs.

Eukaryotic RNA can be modified post-transcriptionally by more than 100 chemical modifications [16]. Among them, N6-methyladenosine (m6A) methylation modification is identified as the most abundant modification [17]. Based on the bulk RNA-seq data analysis from EGFR inhibitor resistant TNBC patient-derived xenografts (PDXs) model, we found m6A binding protein family, insulin-like growth factor 2 mRNA-binding proteins (IGF2BPs), were significantly overexpressed after EGFR inhibitor treatment and IGF2BP3 showed the highest. IGF2BP3 is proved to be expressed specifically high in TNBC [18], and join the differentiation, proliferation, metastasis and other processes of TNBC [19, 20]. It binds extensively and mediates numerous RNAs degradation, splicing and translation by recognizing m6A modifications on targeted mRNAs [21]. Based on the extensive regulatory role of IGF2BP3 in post-transcriptional modification and its positive response to EGFR inhibitor resistance, we hypothesized that IGF2BP3 might serve as a potential therapeutic target to enhance the efficacy of EGFR inhibitors in TNBC.

In the present study, we found IGF2BP3 was overexpressed in EGFR inhibitor resistant TNBC tumor and co-regulated RTKs in an m6A-dependent manner. This functional property may effectively address the poor efficacy of single-agent EGFR inhibitor therapy in TNBC. Following rigorous validation of the specific binding motif between IGF2BP3 and RTK mRNAs, we performed supercomputer-based unbiased computational docking across a small-molecule compound library to search potential IGF2BP3-targeted drug. Through hierarchical screening based on docking scores, a flavonoid compound cynaroside was ultimately identified as a potent inhibitor disrupting the IGF2BP3-RTK mRNA interaction. Validated by in vitro and in vivo experiments, cynaroside effectively hindered tumor progression and improved the therapeutic efficacy of EGFR inhibitor in TNBC. Moreover, we identified a dual-target EGFR/MET inhibitor amivantamab alternative to EGFR single-target inhibitors in combination with cynaroside, provided a practical solution for the precise therapy of TNBC.

## Materials & methods

### Public databases and analysis methods

The Bulk RNA-seq data in TNBC human PDX after erlotinib resistant were acquired from the Gene Expression Omnibus (GEO) database (ID: GSE189257). IGF2BP3 expression data and multiple tyrosine kinase inhibitor (TKI) drug sensitivity area under concentration-time curve (AUC) data of breast cancer cell lines were obtained from GDSC database. Transcriptome sequencing data for 142 TNBC and 695 non-TNBC clinical samples were obtained from the cancer genome atlas (TCGA) database. Differential gene analysis was performed using the R package ‘limma’, with |logFC| > 1 and *P*.adj < 0.001 as the judgment thresholds. Protein expression data for 180 breast cancer tissues were obtained from proteomic data commons (PDB) database and the correlation analysis between IGF2BP3 and RTKs in breast cancer were calculated with the R package ‘corrplot’.

### Cell culture and tissue specimens

Human breast epithelial cell line MCF-10 A (RRID: CVCL_0598) ; TNBC cell lines: MDA-MB-231 (RRID: CVCL_0062), BT549 (RRID: CVCL_1092); luminal breast cancer cell lines: MCF-7 (RRID: CVCL_0031), ZR-75-1 (RRID: CVCL_0588); HER2- breast cancer cell lines: SK-BR-3 (RRID: CVCL_0033), BT474 (RRID: CVCL_0179) were purchased from American Type Culture Collection (ATCC, Manassas, USA) and authenticated by STR profiling. Cells were cultured in high glucose DMEM (Wisent, Nanjing, China) and supplemented with 10% fetal bovine serum, 100 µg/ml penicillin-streptomycin (Hyclone, Logan, USA). Cell culture incubator maintained at 37 °C, 5% CO_2_. TNBC patients tissue samples were collected after surgery from Jiangsu Breast Disease Center, the First Affiliated Hospital with Nanjing Medical University for immunohistochemistry (IHC). Such conductswere reviewed and permitted by the Institutional Ethics Committee of the First Affiliated Hospital of Nanjing Medical University (2024-SR-582). IHC score was conducted using the following criteria: staining intensity (grade 0–4) and percentage of positive cells (grade 0–4).

### Breast cancer model in mice

BALB/c nude mice (aged 4–6 weeks, female) were purchased from (Gempharmatech, Nanjing, China). For subcutaneous inoculation, 5 × 10^6^ MDA-MB-231 cells with 100 µl phosphate buffered saline (PBS) were injected subcutaneously adjacent to the mice mammary fat pads and tail vein. When the tumors volume was about 0.05–0.06 cm^3^, all mice were divided into 5 groups and treated with PBS, cynaroside (MedChemExpress, New Jersey, USA), amivantamab (TargetMol, Boston, USA) and cynaroside+amivantamab respectively. Cynaroside were given by intraperitoneal injection at 50 mg/kg every two days and amivantamab were injected intravenously at 30 mg/kg every four days. The growth of mice weights and tumors volume were recorded every 3 days. After 2 weeks, the tumors and major organs were sold out, fixed with 4% paraformaldehyde, and embedded in paraffin, sectioned for hematoxylin-eosin (H&E) staining and IHC staining afterwards. Plasma was also extracted and analyzed using the automated biochemical analyzer (Rayto, Shenzhen, China) to measure alanine aminotransferase (ALT), aspartate aminotransferase (AST), urea (UREA), creatinine (CREA), creatine kinase (CK), lactate dehydrogenase (LDH), glucose (GLU), and triglycerides (TG). All experiments involving animals were approved by the Animal Ethics Committee of Nanjing Medical University (IACUC-2206017).

### Lentivirus and plasmids transfection

The lentiviral shRNAs and lentiviral plasmid of IGF2BP3 were used as our previous study [18]. Truncated expression constructs of IGF2BP3 with Flag and green fluorescent protein (GFP) tags were produced by (Corues, Nanjing, China). Plasmids were transfected with Lipofectamine 3000 (Invitrogen, Carlsbad, USA). The sequences of plasmids were listed in Table S1.

### RNA extraction and quantitative real-time PCR (qRT-PCR)

Total RNA was isolated from cells using TRIzol™ reagent (Takara, Kyoto, Japan) following the manufacturer’s protocol. Reverse transcription of mRNA into complementary DNA (cDNA) was performed with HiScript^®^ Q RT SuperMix (Vazyme, Nanjing, China). qRT-PCR was carried out on a LightCycler^®^ 480 System (Roche Diagnostics, Basel, Switzerland) using SYBR Green detection.

### Western blot

Cells were lysed in ice-cold RIPA buffer (Beyotime, Shanghai, China) supplemented with protease and phosphatase inhibitors (MedChemExpress, New Jersey, USA). Protein concentrations were quantified using a bicinchoninic acid (BCA) assay kit (Beyotime, Shanghai, China). Equal amounts of protein were resolved by SDS-PAGE and transferred onto polyvinylidene difluoride membranes (Millipore, Bedford, USA). Membranes were blocked with 5% bovine serum albumin in Tris buffered saline tween for 1 h at room temperature, followed by overnight incubation at 4 °C with primary antibodies. After three TBST washes, membranes were incubated with horseradish peroxidase (HRP)-conjugated secondary antibodies (Cell Signaling Technology, Boston, USA) for 1 h at room temperature. Protein bands were visualized using Immobilon^®^ Western Chemiluminescent HRP Substrate (Millipore, Bedford, USA). Gray values were quantified using ImageJ software (https://imagej.net/) and the relative levels of targeted protein were normalized by the control group. Primary antibodies used in this study were listed in Table S2.

### RNA immunoprecipitation (RIP) and methylated RNA immunoprecipitation (MeRIP)

For RIP, cells were lysed in RIP-specific lysis buffer (Millipore, Bedford, USA) and incubated overnight at 4 °C with 5 µg of anti-IGF2BP3 antibody or control IgG (Beyotime, Shanghai, China). RNA-protein complexes were immunoprecipitated using Protein A/G magnetic beads (MedChemExpress, New Jersey, USA) for 2 h at 4 °C. Following extensive washing with RIP wash buffer, bound RNAs were eluted, purified using the RNeasy Mini Kit (Qiagen, Hilden, Germany), reverse-transcribed into cDNA with HiScript^®^ Reverse Transcriptase (Vazyme, Nanjing, China), and quantified by qRT-PCR.

For MeRIP, total RNA was extracted using TRIzol™ reagent (Takara, Kyoto, Japan). RNA fragmentation was performed with RNA Fragmentation Buffer (Millipore, Bedford, USA) to generate ~ 100-nucleotide fragments. Fragmented RNA was incubated for 2 h at 4 °C with anti-m6A antibody using the Magna MeRIP™ m6A Kit (Millipore, Bedford, USA), followed by immunoprecipitation with Protein A/G magnetic beads. Enriched m6A-modified RNAs were eluted, reverse-transcribed, and analyzed by qRT-PCR as described above. Results were normalized to input RNA levels. Primer sequences for target transcripts were provided in Table S3.

### RNA stability analysis

5 × 10⁵ cells were seeded into 6-well plates and cultured for 24 h after adding cynaroside (10 µM). Actinomycin D (ActD, 5 µg/ml) was then added at 0, 2, 4, 6, 8, and 10 h. Total RNA was extracted at appropriate time points. qRT-PCR was used to detect the degradation rates of EGFR and MET RNA.

### Co-immunoprecipitation(co-IP)

Protein A/G magnetic beads (MedChemExpress, New Jersey, USA) were conjugated with either anti-EGFR primary antibody or IgG isotype control by incubation in IP buffer for 2 h at 4 °C with gentle rotation. Cells and tumor were lysed in ice-cold IP lysis buffer (Beyotime, Shanghai, China) supplemented with protease/phosphatase inhibitors (1×) for 30 min on ice and incubated with the antibody-conjugated beads overnight at 4 °C. Beads were subsequently washed five times with high-salt wash buffer. Bound protein complexes were eluted using 1× loading buffer (95 °C, 10 min) and subjected to downstream immunoblotting analysis.

### RNA pull-down Assay

Biotinylated RNA probes were synthesized and purified by high performance liquid chromatography (Sangon, Shanghai, China). Streptavidin-conjugated agarose magnetic beads (Smart-Lifesciences, Changzhou, China) were pre-washed with lysis buffer and incubated with the biotinylated probes for 1 h at room temperature. Cells were lysed in ice-cold lysis buffer containing RNase and protease inhibitors. Lysates were clarified by centrifugation and supernatants were incubated with the RNA probe-bound beads overnight at 4 °C. After being washed five times, bound RNA-protein complexes were eluted using biotin competition buffer and subjected to protein extraction.

### Computer aided drug screening and molecular docking

Firstly, the crystal complex structure of KH 1–2 domain in IGF2BP3 was obtained from the PDB database (PDB ID:6GQE). The hydrophobic pocket connected to RNA was selected in Schrödinger software (https://www.schrodinger.com/). Then, using Glide docking algorithm, the small molecule compounds in ChemDiv database were virtually screened with the above hydrophobic pockets, and the top 1,000 compound molecules were screened in three stages (HTVS, SP and XP) to obtain the top 10 ranked candidate compounds. The downloaded structures of proteins and small molecule compounds were processed with PyMOL 2.3.0 software (https://pymol.org/) to remove water molecules, label docking sites and graph.

### Cytotoxicity assay

Cells were seeded in 96-well plates (1 × 10^4^ cells/well) and grew in complete medium containing different concentrations of cynaroside (3–15 µM), amivantamab (15–1000 µg/ml), linsitinib (1.25-20 µM), erdafitinib (1.25-20 µM) and equivalent doses cynaroside+amivantamab, cynaroside+linsitinib, cynaroside+erdafitinib for 48 h, respectively. The cell viability was further tested by Cell Counting Kit-8 (CCK-8) kit (Vazyme, Nanjing, China) following the manufacturer’s guidelines. CompuSyn (https://www.combosyn.com/) and Prism (https://www.graphpad.com/) software were used to calculate the combination index (CI) and half maximal inhibitory concentration (IC50).

### Colony formation assay

For colony formation assay, cells were seeded in six-well plates with 1000 cells/well for attachment and incubated with cynaroside (6µM), amivantamab (500 µg/ml), cynaroside+amivantamab for 14 days, equal amount of dimethyl sulfoxide (DMSO) was added as control. The resulting colonies were stained with crystal violet solution and then the colonies were photographed (Nikon, Tokyo, Japan). Image J software (https://imagej.net/) was used to measure the number of clones.

### Transwell assay

Placing the cell culture chamber (Corning, Corning, USA) into a 24-well plate. Simultaneously, seeding 3 × 10⁵ cells in the upper well using serum-free DMEM supplemented with the drug. At the same time, filling the lower well with 600 µl of medium containing 10% FBS as the cell migration catalyst. After incubation for 24–48 h, successfully transited cells are stained with 1% crystal violet. Photographing of migrating cells under a microscope (Nikon, Tokyo, Japan).

### 3D cell sphere invasion assay

After centrifugation, 1000 cells were suspended and cultured in a 96-well ultra-low-binding plate (labselect, Suzhou, China) for 3 days to form cell spheroids. Matrix gel (Corning, Corning, USA) was then added and incubated at 37 °C for one hour to solidify. DMEM medium containing various drugs was added to the upper layer of the matrix gel and cultured for 48 h. Photographs were taken under a 10x objective lens. ImageJ software was used to calculate the ratio of invasive area to original spheroid area.

### Microscale thermophoresis (MST) assay

Plasmid constructs encoding full-length IGF2BP1, IGF2BP2, IGF2BP3 and truncated variants (ΔRRM1-2: 1-195; ΔKH1-2: 196–344; ΔKH3-4: 345–579) of IGF2BP3 fused to GFP were transfected into HEK-293T cells. Cells were lysed via sonication in ice-cold PBS supplemented with protease inhibitors. Small-molecule compounds were serially diluted in 16-point gradients starting from an initial concentration of 50 mM. GFP-tagged protein lysates were mixed 1:1 (v/v) with each diluted compound in MST-compatible capillaries (NanoTemper, Munich, Germany). Thermophoretic mobility was measured using a Monolith NT.115 Pico MST instrument (NanoTemper, Munich, Germany). Fluorescence redistribution in response to localized temperature gradients was recorded over time. Binding curves were generated using MO.Affinity analysis software (https://shop.nanotempertech.com/), and dissociation constants (KD) were calculated via nonlinear regression fitting of the normalized fluorescence change against compound concentrations.

### Statistical analysis

Data analysis and visualization were performed using GraphPad Prism software (https://www.graphpad.com/). Intergroup differences were assessed using unpaired Student’s t-test (two-group comparisons) or one-way ANOVA (multi-group comparisons). All experiments were conducted in three biologically independent replicates, and data were presented as mean ± SEM. Statistical significance was defined as *P* < 0.05 (*), *P* ≤ 0.01(**), *P* ≤ 0.001(***).

## Result

### High expression of IGF2BP3 could restrict the drug sensitivity of EGFR inhibitors in TNBC

**Fig. 1:**
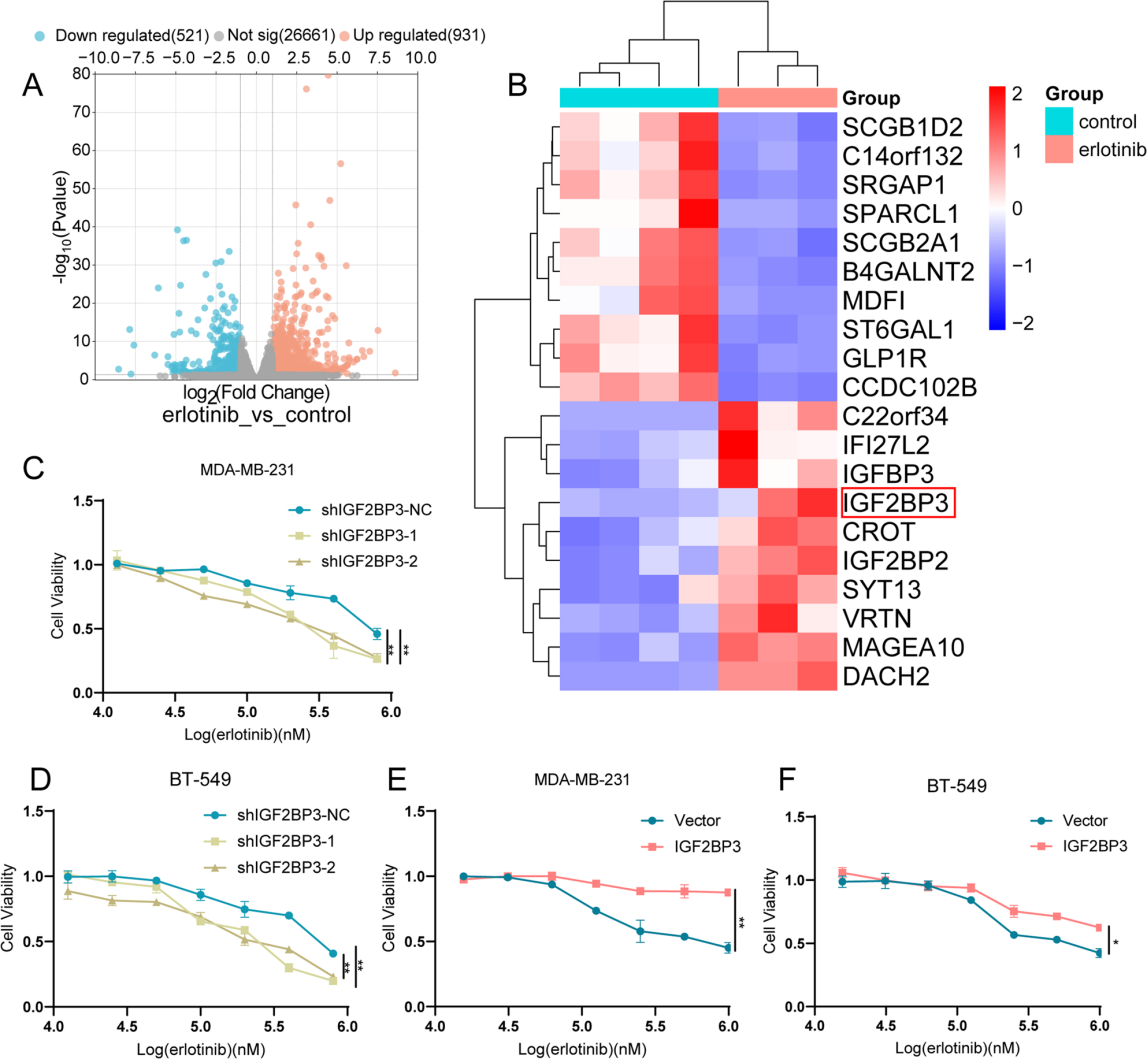
High IGF2BP3 expression may limit erlotinib efficacy in TNBC. **A** Volcano plot demonstrated 931 genes upregulated and 521 genes downregulated after erlotinib treatment. **B** Cluster plot showed the top 20 genes with the most significant differences between the erlotinib-treated group and the control group. **C**-**D**. MDA-MB-231 (**C**) and BT-549 (**D**) cells after IGF2BP3 knockdown were treated with graded concentrations of erlotinib. The cytotoxic effect of erlotinib were increased after IGF2BP3 knockdown. **E**-**F**. The cytotoxic effect of erlotinib were decreased after IGF2BP3 overexpression in MDA-MB-231 (**E**) and BT-549 (**F**) cells

Secondary analysis of bulk RNA-seq data from the GEO dataset (ID: GSE189257) compared transcriptional profiles of erlotinib-treated (9-week regimen) versus untreated TNBC patient-derived xenograft (PDX) tissues (*n* = 3 vs. *n* = 4) was conducted (Fig. S1A). Using |log_2_FC|>1, *P.*adj < 0.05 as the threshold, we identified 931 genes with elevated expression and 521 genes with decreased expression in the erlotinib-treated group (Fig. 1A). Genes were ranked based on log_2_FC and *P.*adj to identify the top 20 genes with the most significant differences. Among them, the expression of the m6A-binding protein IGF2BP family was found to be significantly elevated in erlotinib treated tissues, with the most significant difference was IGF2BP3 (Fig. 1B).

We have previously constructed MDA-MB-231 and BT-549 cell lines that stably overexpressed and knocked down IGF2BP3 [18]. Following the treatment with identical concentration gradients of erlotinib, the viability of IGF2BP3 knockdown cells decreased by 50% at an approximate concentration of 200 µM, whereas the viability of control cells remained at 80% under the same conditions (Fig. 1C-D). Conversely, IGF2BP3 overexpression cells maintained better survival rates after the treatment of the identical concentrations of erlotinib. Even under higher concentration of erlotinib at 1 mM, MDA-MB-231 cells and BT-549 cells after IGF2BP3 overexpression still survived 87% and 62%, respectively (Fig. 1E-F). These results implicated that high expression of IGF2BP3 could limit the therapeutic efficacy of EGFR inhibitors in TNBC.

### IGF2BP3 could co-regulate RTKs in an m6A-dependent manner to restrict drug sensitivity for EGFR inhibitors in TNBC

**Fig. 2:**
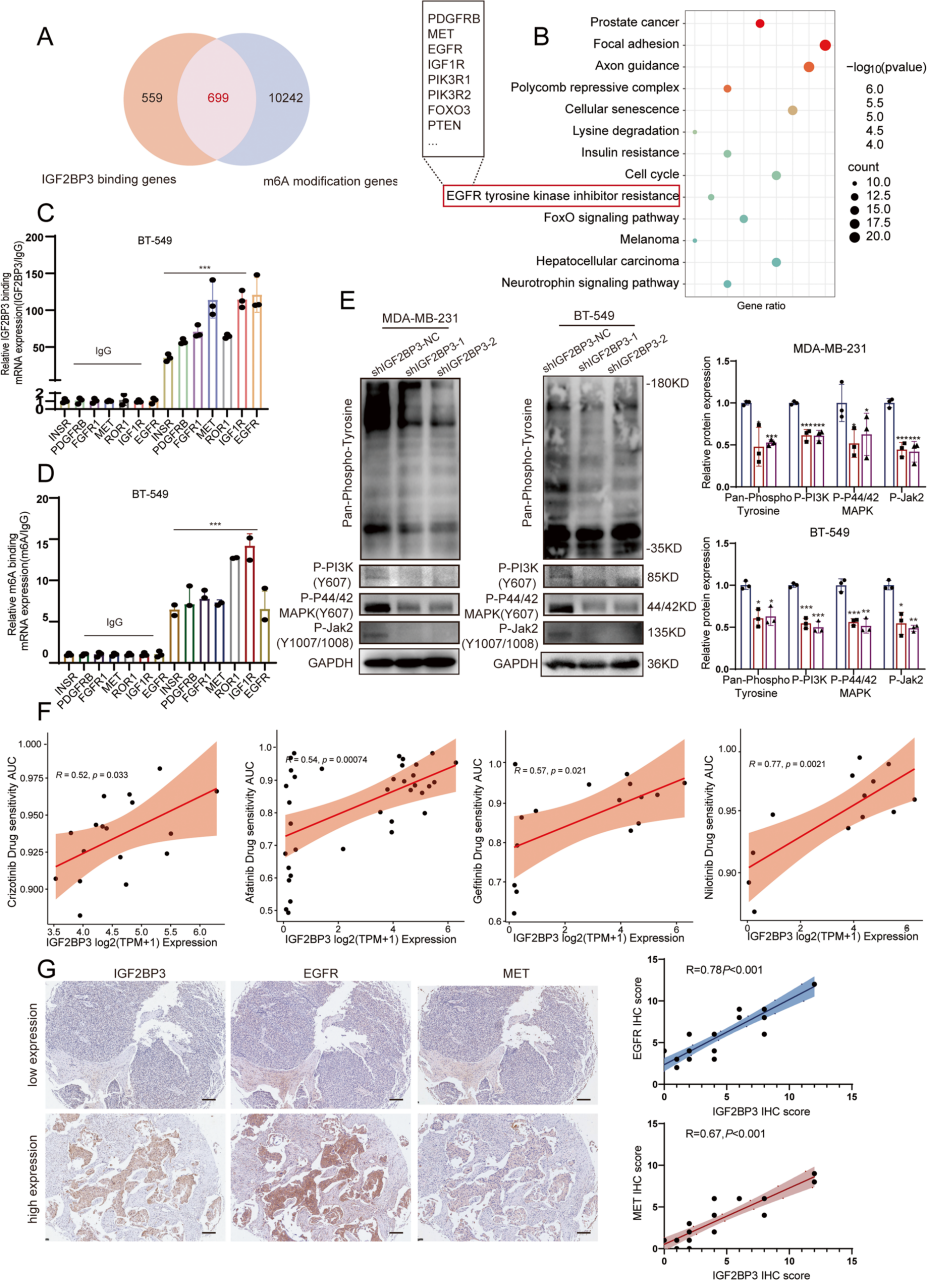
Multiple RTKs were co-regulated by IGF2BP3 in an m6A-dependent manner. **A** venn diagram showed the intersection genes of IGF2BP3 RIP- seq and MeRIP-seq. **B** KEGG pathway analysis demonstrated genes intersected by RIP-seq and MeRIP-seq. **C **RIP-qRT-PCR clarified the direct connection between IGF2BP3 and RTKs mRNA. **D** MeRIP-qRT-PCR verified the direct m6A modification in RTKs mRNA. **E** decreased levels of pan-tyrosine and PI3K/MAPK/JAK2 phosphorylation were detected after IGF2BP3 knockdown. The relative gray values of targeted protein were normalized by the control group. **F** A correlation analysis of IGF2BP3 expression and drug sensitivity AUC in breast cancer cell lines from the GDCS database showed that high IGF2BP3 expression was associated with reduced sensitivity to multiple TKI drugs. **G** a correlation analysis of IGF2BP3, EGFR and MET expression in human TNBC tissue. Quantification using IHC score (n=30)

In previous studies, the gene profile regulated by IGF2BP3 in an m6A-dependent manner was systematically characterized through integrated IGF2BP3 RIP-seq and m6A-RIP-seq (MeRIP-seq) in TNBC cell (Fig. 2A) [18]. Through KEGG pathway enrichment of the overlapping genes derived from the above two sequencing datasets, we found that IGF2BP3 targeted genes were highly enriched in the EGFR tyrosine kinase inhibitor resistance pathway, which contained several RTK family members (Fig. 2B). RIP/MeRIP-qPCR confirmed that IGF2BP3 could bind to multiple RTK transcripts, including EGFR, MET and FGFR1 etc. in an m6A-dependent manner (Fig. 2C-D). Meanwhile, global tyrosine phosphorylation level the critical downstream signaling components like PI3K, MAPK, and JAK2 phosphorylation level in TNBC cells were elevated after IGF2BP3 overexpression and reduced after IGF2BP3 knocking down (Fig. 2E, Fig. S1B-C). Subsequently, we collected IGF2BP3 expression data and TKI drug sensitivity AUC data in a variety of breast cancer cell lines through the GDSC database. The Pearson correlation analysis revealed that high IGF2BP3 expression was correlated with elevated AUC values for EGFR/HER2 inhibitors (gefitinib, afatinib) and MET inhibitors (crizotinib) (Fig. 2F). Concurrently, we collected 30 cases of TNBC tissue and verified the expression levels of IGF2BP3, EGFR, and MET through IHC. Results demonstrated that IGF2BP3 expression exhibited a high positive correlation with EGFR and MET, with correlation coefficients of 0.78 and 0.67, respectively (Fig. 2G). All the evidences implied that IGF2BP3 could co-regulated RTKs in an m6A-dependent manner to restrict drug resistance of EGFR inhibitors in TNBC.

### MET-EGFR heterodimer, mainly bound to IGF2BP3 KH1-2 domain as IGF2BP3 core-regulated RTKs in TNBC

**Fig. 3:**
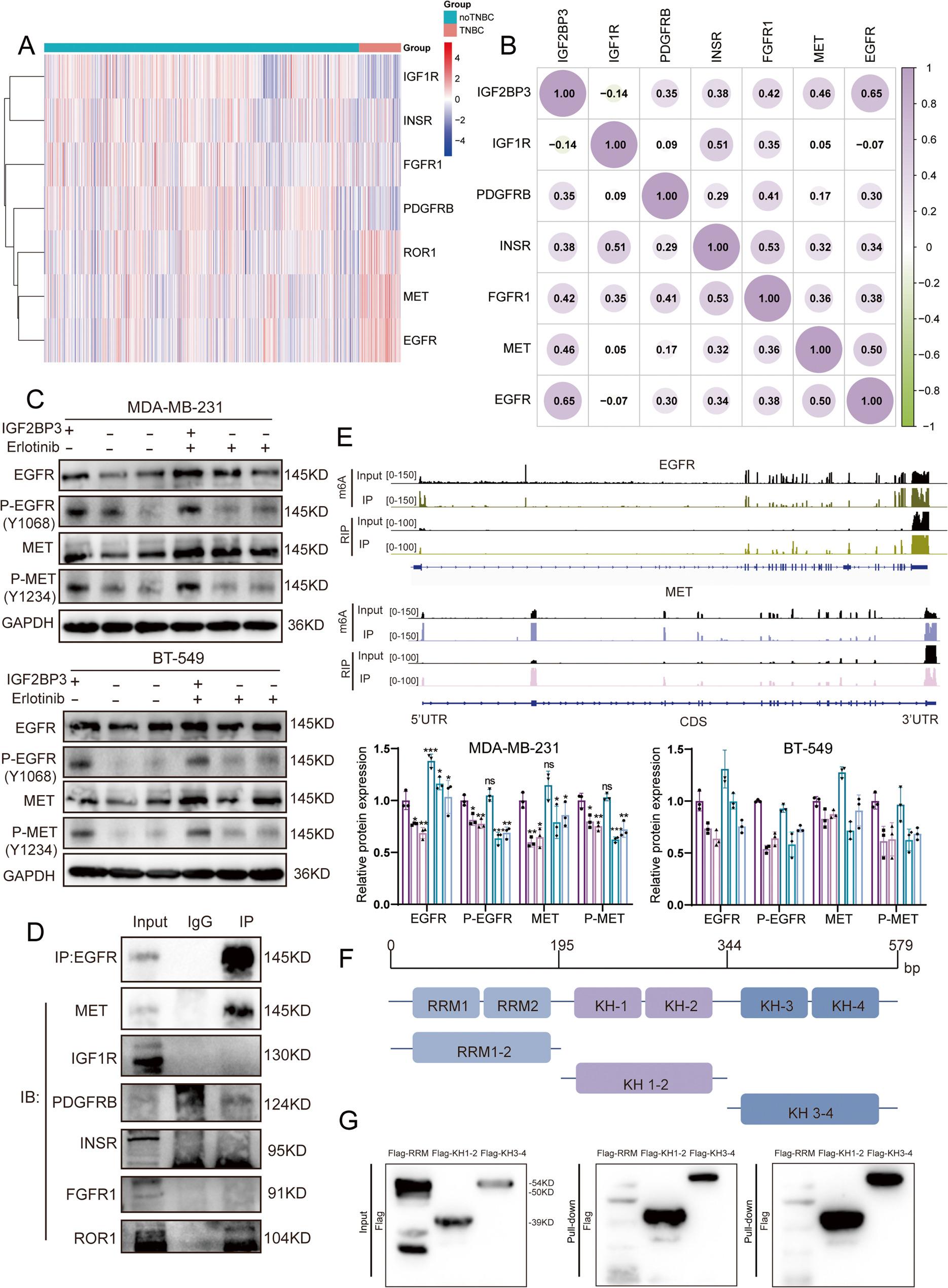
As IGF2BP3 core-regulated RTKs mRNAs in TNBC, EGFR, MET mRNAs mainly bound to IGF2BP3 KH1-2 domain. **A** Cluster heatmap showed the expression of IGF2BP3 binding RTKs in TNBC tissues compared with no-TNBC tissues. Data from TCGA database. **B** Protein expression correlation analysis in breast cancer tissues between IGF2BP3 and IGF2BP3 binding RTKs. Data from PDC database. **C** The expression of EGFR and MET and their phosphorylation level in TNBC cells were detected by western blot after the knockdown of IGF2BP3 with or without erlotinib. **D** Western blot after co-IP showed the interactions between EGFR and IGF2BP3 regulated RTKs. **E** Distribution of IGF2BP3-binding peaks by RIP-seq and m6A peaks by MeRIP-seq across EGFR/MET mRNA. **F** Plasmids were constructed by dividing IGF2BP3 into three segments based on domains (pcDNA3.1-GFP-Flag-RRM1-2, pcDNA3.1-GFP-Flag-KH1-2, pcDNA3.1-GFP-Flag-KH3-4). **G** Pull-down assay was conducted using biotin probes to reveal the binding of IGF2BP3 KH1-2 and KH3-4 domain on EGFR/MET mRNA

In order to identify the main RTK targets by IGF2BP3 in TNBC, we collected the expression profiles of IGF2BP3 binding RTKs from TCGA breast cancer database and categorized the database samples into TNBC and no-TNBC groups. Among the RTKs, MET and EGFR were found highly expressed in TNBC than no-TNBC group (Fig. 3A). Correlation analysis of protein expressions between IGF2BP3 and IGF2BP3 binding RTKs in breast cancer tissues from PDC database further revealed the strongest co-expression between IGF2BP3 and EGFR/MET proteins (Fig. 3B). After IGF2BP3 knockdown in MDA-MB-231 and BT-549 cells, total EGFR and MET proteins expression and their related phosphorylation level were also decreased (Fig. 3C). Furthermore, co-IP using anti-EGFR antibody in MDA-MB-231 lysates confirmed EGFR could form heterodimers with MET and ROR1, with the MET-EGFR heterodimer being the most prominent. (Fig. 3D). In summary, MET-EGFR heterodimer was considered to be the core IGF2BP3-regulated RTKs in TNBC.

RIP/MeRIP-seq peak calling was then performed to elucidate the binding domains of IGF2BP3 with EGFR and MET mRNAs. It revealed that IGF2BP3 protein localized at the 5′- and 3′-untranslated regions (UTRs) of EGFR and MET mRNA, characterized by the presence of the highly repetitive m6A sequence “RRACH” (Fig. 3E). RNA probes containing these motifs were synthesized for in vitro RNA pull-down assays. Because IGF2BP3 comprised of six structural domains [22], we engineered GFP-Flag-tagged IGF2BP3 truncation constructs (ΔRRM1-2: 1-195; ΔKH1-2: 196–344; ΔKH3-4: 345–579) and expressed them in HEK293T cells respectively (Fig. 3F). Finally, RNA pull-down experiments were performed using the above RNA probes and HEK293T cell lysates. Western blot confirmed that EGFR and MET mRNA could bind to the IGF2BP3 KH1-4 domain, with a preference in KH1-2 domain (Fig. 3G).

### Cynaroside was screened as a small molecule inhibitor for competitively disrupting RNA-protein interactions

**Fig. 4:**
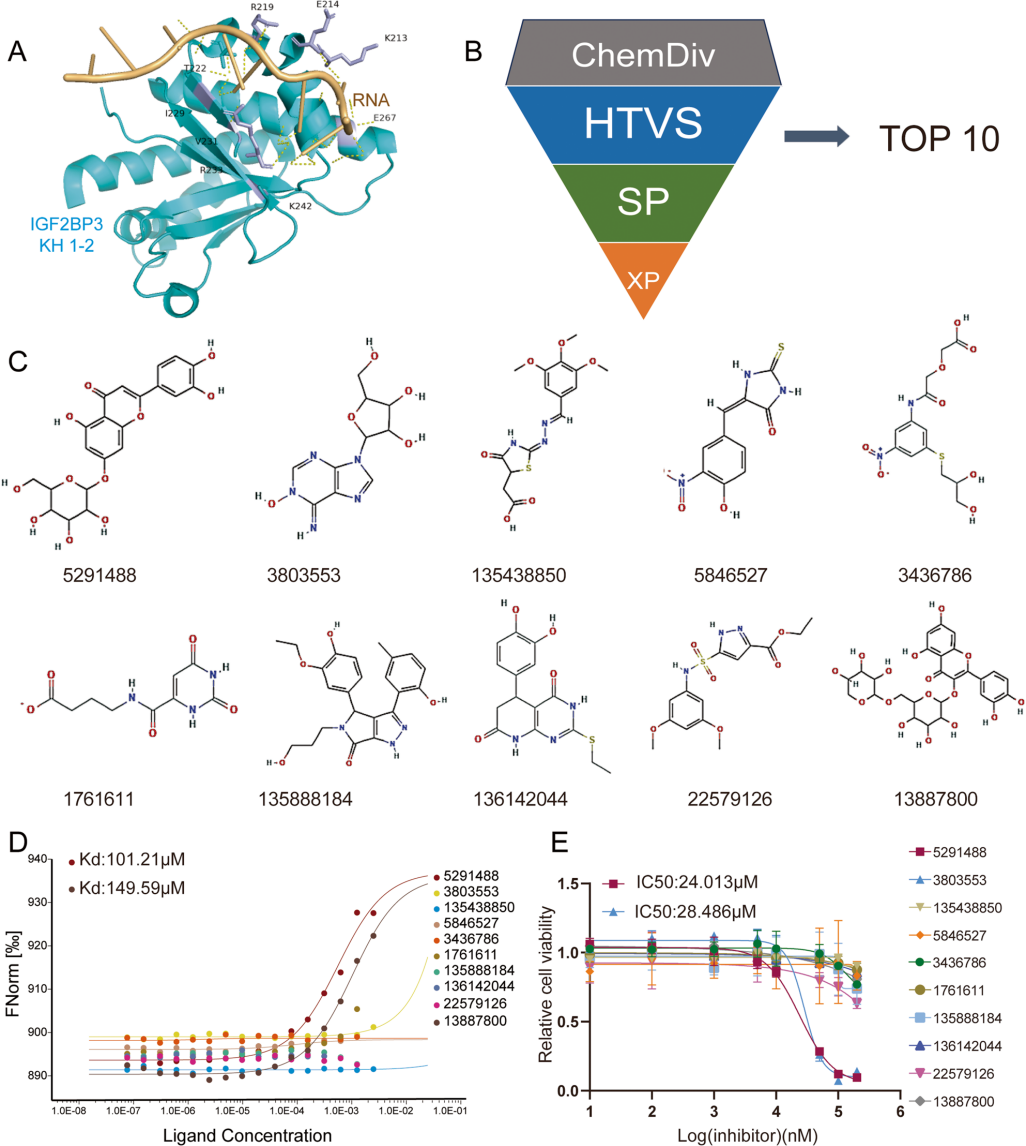
5291488 was screened effectively targeting IGF2BP3 and killing TNBC cells. **A** Diagram of the IGF2BP3 KH1-2 domain and RNA virtual docking pattern. **B** The small molecule compounds were screened through three levels of high-throughput (HTVS), standard precision (SP) and extra precision (XP). **C** Structural formulae of the top ten small molecule compounds. The images were from the Pubchem database. **D** MST experiments demonstrated the binding ability of the IGF2BP3 protein to 10 small molecule compounds. **E** MDA-MB-231 cells were treated with different dosages of the 10 small molecule compounds for 24 h, respectively. CCK-8 assay was used to detect the cell viability

Based on the above structural domains, we retrieved the crystal structure of the IGF2BP3 KH1-2 domain (ID:6GQE) in complex with RNA (ID:7YEY) from the PDB database and imported it into the Schrödinger software. A hydrophobic pocket comprising residues Lys-213, Arg-219, Thr-222, Arg-233, Lys-242 and Glu-267, which interacts with RNA, was selected for virtual screening (Fig. 4A). Utilizing supercomputing resources, we performed Glide docking algorithms to screen the ChemDiv compound library against this hydrophobic pocket (Fig. 4B). The top 1,000 compounds ranked by binding affinity underwent three sequential stages of docking refinement (high-throughput, standard precision, and extra precision), yielding 10 top-ranked candidates for experimental validation (Fig. 4C). The GFP-6×HIS-IGF2BP3 full-length plasmid was transfected into HEK293T cells, and the cell lysate mixed with small molecule compounds for in vitro docking experiments. MST assays demonstrated the binding of compounds 5,291,488 and 13,887,800 to IGF2BP3, with KDs of 101.21 µM and 149.59 µM, respectively (Fig. 4D). Furthermore, by testing the ten compounds with different concentration gradients in MDA-MB-231 cells, only 5,291,488 and 3,803,553 exhibited cytotoxic effects, with IC50 values of 24.013 µM and 28.486 µM, respectively (Fig. 4E). Together, these findings established 5,291,488 as a small-molecule IGF2BP3 inhibitor possessing anti-TNBC activity.

**Fig. 5:**
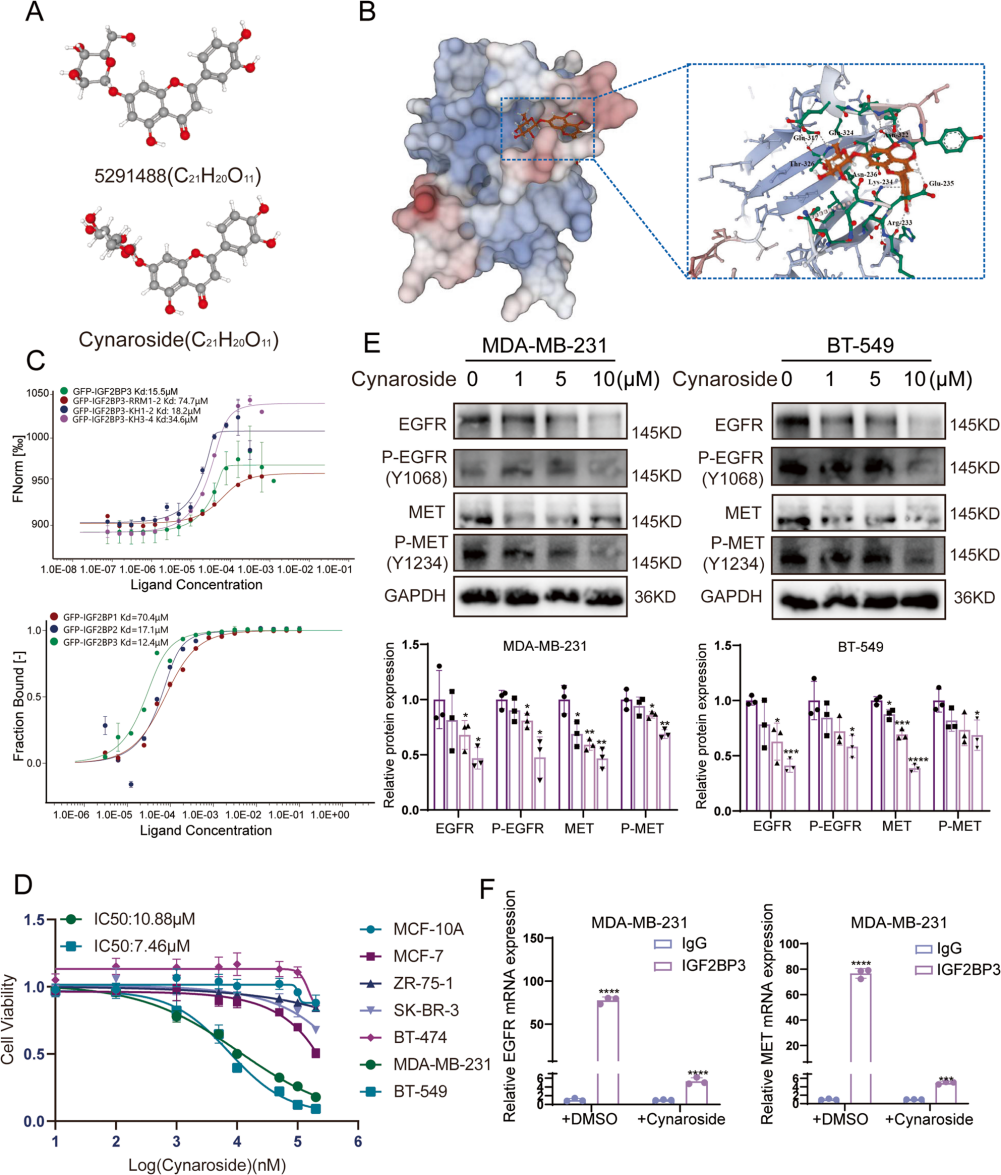
Cynaroside was a better small molecule inhibitor targeting IGF2BP3 KH1-2 domain than 5291488. **A** Ball-and-stick model of 5291488 and cynaroside. The images were from the Pubchem database. **B** Diagram of the IGF2BP3 KH1-2 domain and cynaroside virtual docking pattern. **C** MST assay demonstrated the binding ability of IGF2BP1/IGF2BP2/IGF2BP3 and the different domains of IGF2BP3 protein to cynaroside. **D** Human breast epithelial cell line MCF-10A; TNBC cell lines: MDA-MB-231, BT549; luminal breast cancer cell lines: MCF-7, ZR-75-1; HER2- breast cancer cell lines: SK-BR-3, BT474 were treated with gradient doses of cynaroside for 24 h, respectively. CCK-8 was used to detect the cell viability. **E** MDA-MB-231 and BT-549 cells were treated by cynaroside. EGFR and MET protein and their phosphorylation levels was demonstrated by western blot. **F** RIP-qRT-PCR showed the direct connection between IGF2BP3 and EGFR/MET mRNA and the binding capacity decreased after cynaroside treatment. Data were shown as the mean ± SEM of three replicates; *P < 0.05

Although 5,291,488 potently targeted IGF2BP3 and exhibited cytotoxic effects against TNBC, its high KD and IC50 value resulted in a narrower therapeutic window and increased dose-limiting toxicities. So, we screened the structural analogs of 5,291,488 via the PubChem database and identified cynaroside as a promising candidate to substitute 5,291,488. Cynaroside shares the same molecular formula as 5,291,488 and only differs in the conformation of a cyclohexane moiety: 5,291,488 adopts a boat conformation, whereas cynaroside exhibits a chair conformation, which is usually more stable at room temperature (Fig. 5A). Virtual docking using Schrödinger software predicted that cynaroside effectively engaged the RNA-binding hydrophobic pocket within the IGF2BP3 KH1-2 domain (Fig. 5B). MST assays demonstrated a 10-fold enhancement in the binding affinity of cynaroside to full-length IGF2BP3 compared to 5,291,488, with the highest affinity of KH1-2 domain. Moreover, cynaroside also exhibited binding capacity toward other members of the IGF2BP family, with IGF2BP3 remaining the strongest binding partner among them (Fig. 5C). Treatment of various breast cancer subtype cells and normal mammary epithelial cells with a concentration gradient of cynaroside revealed that cynaroside exerted selective cytotoxic effects against TNBC cells. Moreover, the IC50 value of cynaroside was half that of 5,291,488 (Fig. 5D). Based on our previous study, we demonstrated that IGF2BP3 primarily modulates MET expression by regulating mRNA translation initiation [23]. qRT-PCR and mRNA stability assays confirmed that neither the RNA expression (Fig. S2A) nor stability of EGFR and MET mRNA was altered following the addition of cymaroside (Fig. S2B-C). However, western blot confirmed a dose-dependent reduction in total EGFR and MET protein levels and their phosphorylation levels following cynaroside treatment in MDA-MB-231 and BT-549 cells (Fig. 5E). This confirmed that cynaroside effectively inhibits the protein expression of EGFR/MET rather than their RNA expression. Furthermore, RIP-qPCR demonstrated significantly diminished binding capacity of IGF2BP3 to EGFR/MET mRNA in cynaroside-treated TNBC cells (Fig. 5F). Collectively, these findings identified cynaroside as a more promising small molecule inhibitor that exhibited enhanced binding capabilities with IGF2BP3 and potent anti-TNBC activity.

### Cynaroside synergized with amivantamab could effectively inhibit TNBC cell growth and metastasis

To investigate whether cynaroside could overcome erlotinib resistance in TNBC, MDA-MB-231 and BT-549 cells with treated with cynaroside, erlotinib alone, or their combination. The combination of cynaroside and erlotinib resulted in a 10% increase in inhibiting TNBC growth, compared to cynaroside alone at a concentration of 10 µM, at which erlotinib was not cytotoxic (Fig. S3A-B). Colony formation assay also showed better inhibition of cell proliferation in the cynaroside+erlotinib group compared to the single agent treatment group (Fig. S3C-D). These results indicated that cynaroside effectively sensitized TNBC cells to erlotinib. However, even in combination with cynaroside, erlotinib required an administered concentration of 10 µM or more for limited antitumor efficacy, thereby restricting its potential for clinical translation.

**Fig. 6:**
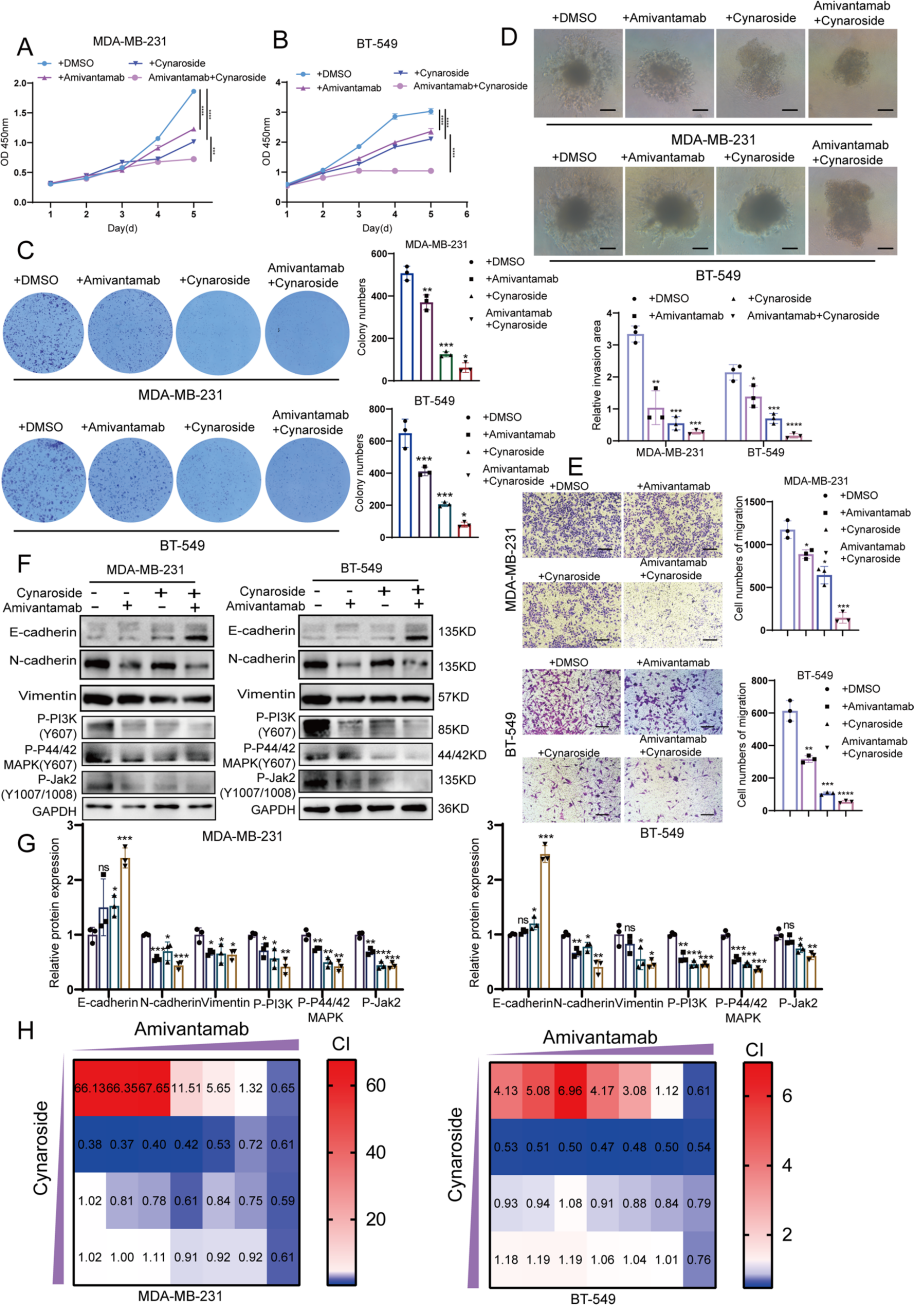
Cynaroside synergized with amivantamab could effectively inhibit TNBC cell growth. **A**-**B** MDA-MB-231 and BT-549 cells were treated with cynaroside (6 μM), amivantamab (500 μg/ml) and cynaroside+amivantamab (6 μM+500 μg/ml). CCK-8 was used to record cell growth curves for 5 days. **C** Representative images and quantifications of cell colonies after cymaroside, amivantamab and cynaroside+amivantamab treatment for 14 days. Data were shown as mean ± SD of three replicates. **D** Following treatment with cynaroside (6 μM) and amivantamab (500 μg/ml) for 48h, either alone or in combination, the 3D spheroid cell invasion assay assessed cellular invasion capacity. The ratio of invasive area to original spheroid area were calculated. Data were shown as the mean ± SD of three replicates; P < 0.05 (*), P≤0.01(**), P≤0.001(***). **E** Migration ability of MDA-MB-231 and BT-549 cells changed by cynaroside (6 μM) and amivantamab (500 μg/ml) were exhibited by transwell assay. Scale bar, 50 μm. **F**-**G** PI3K, P44/42 MAPK and Jak2 phosphorylation levels and E-cadherin, N-cadherin and Vimentin expression were demonstrated by western blot after cynaroside, amivantamab and cynaroside+amivantamab treatment for 48h. Data were shown as the mean ± SD of three replicates; P < 0.05 (*), P≤0.01(**), P≤0.001(***). **H** Growth inhibition and CI of MDA-MB-231 and BT549 cells at different drug concentrations for 48h. Red color indicated competition (CI>1), blue color indicated synergy (0.8 ≤ CI < 0.9: low synergy, 0.6 ≤ CI < 0.8: moderate synergy, 0.4 ≤ CI < 0.6: high synergy, 0.2 ≤ CI < 0.4: strong synergy). The values were shown as CI. Amivantamab concentrations were 15, 31, 62.5, 125, 250, 500, 1000 μg/ ml from left to right, and cynaroside concentrations were 3, 6, 12 and 15 μM from top to bottom

Fortunately, we found that amivantamab could be a potentially superior alternative to erlotinib for TNBC therapy. Amivantamab, an IgG1 bispecific antibody, can disrupt EGFR/MET’s ligands binding at the same time, partly overcomes the problem of poor single-targeted EGFR therapy [24]. Through cell proliferation (Fig. 6A-B) and colony formation assay (Fig. 6C), we found that amivantamab monotherapy already presented antitumor activity in MDA-MB-231 and BT-549 cells. Also, 3D cells sphere invasion assay (Fig. 6D) and transwell assay (Fig. 6E) demonstrated reduced cell migration and invasion capabilities following amivantamab treatment. Moreover, the the epithelial phenotype marker E-cadherin and mesenchymal phenotype markers, N-cadherin, Vimentin and the phosphorylation of critical downstream proteins like PI3K, MAPK, and JAK2 were correspondingly changed after amivantamab treatment (Fig. 6F-G).

Furthermore, we set up the cynaroside+amivantamab group along with the single-agent treatment group. It showed that MDA-MB-231 and BT-549 cells almost stopped growing after treatment with cynaroside in combination with amivantamab (Fig. 6A-C). Furthermore, following combined drug treatment, the cells exhibited a more substantial reduction in their invasive and migratory capabilities (Fig. 6D-E). The phosphorylation of PI3K, MAPK, and JAK2 were further reduced in drug combination group (Fig. 6F-G). Subsequently, to rule out the stacking effect, we designed a concentration gradient based on the IC50 of both agents and evaluated the synergistic effect of cynaroside with amivantamab using CompuSyn modeling. The results showed that cynaroside combined with amivantamab had a moderate-to-strong synergistic interactions (CI < 1) in both cell lines, and the best synergistic effect was achieved at cynaroside 6µM and amivantamab 31 µg/ml. (Fig. 6F-G). Moreover, the synergistic effects of these two drugs both superior to the combined treatment of cynaroside with other TKIs (such as the IGF1R/INSR inhibitor linsitinib and the FGFR family inhibitor erdafitinib) (Fig. S3E-F). Collectively, these findings demonstrated that cynaroside synergized with amivantamab effectively suppressed TNBC cell growth and metastasis.

### Cynaroside in combination with amivantamab safely inhibited TNBC progression in mice

**Fig. 7:**
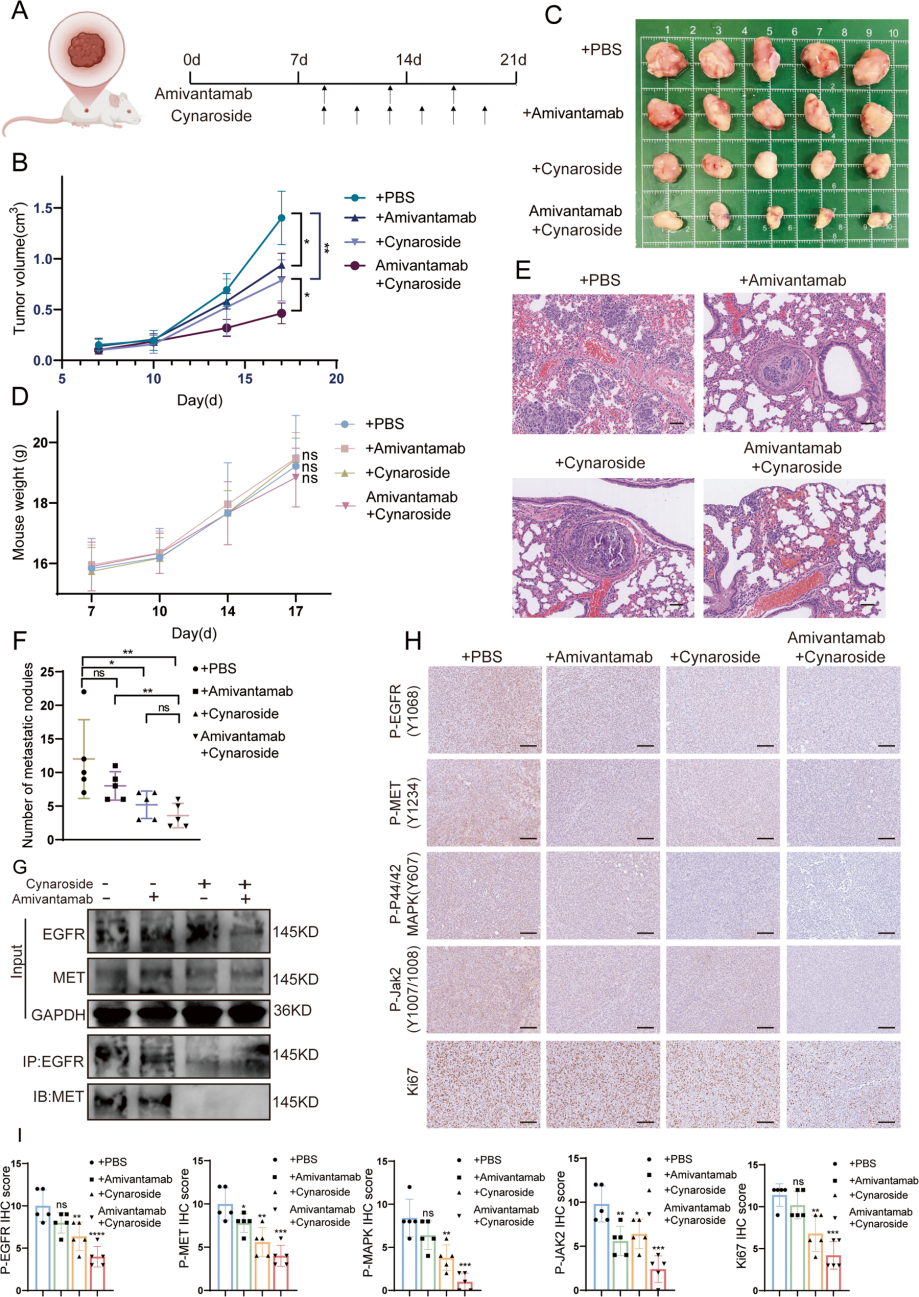
Cynaroside in combination with amivantamab safely inhibited TNBC progression in vivo. **A** After one week of tumor formation (n=20), amivantamab (30 mg/kg) was administered by intravenous injection every four days, cynaroside (50 mg/kg) was administered intraperitoneally every two days for 14 days. **B**-**C** Tumor volume growth curves (**B**) and tumor shots (**C**) demonstrated the effect of cynaroside (n=5), amivantamab (n=5) and cynaroside+amivantamab (n=5) treatment on tumor growth. Data were shown as mean ± SD. **D** Mouse body weight recordings demonstrated the effect of different drug treatment groups on basic health indicators in mice between groups. **E**-**F** Representative images (**E**) and the statistical count for each group (**F**) revealed the lung metastasized tumors following treatment with drugs alone and in combination. HE staining of lung section (n=5) showed the pathological properties of the nodules. Scale bar, 120 μm. **G** Western blot after co-IP showed the interactions between EGFR and MET in tumor tissues from mice in different groups. **H**-**I** The phosphorylation level of EGFR, MET, PI3K, MAPK, Jak2 and Ki67 expression were demonstrated by IHC in TNBC tumor tissue (original magnification, 10×). Scale bar, 120 μm. IHC score in different groups were calculated by on way ANOVA (n=5). P < 0.05 (*), P≤0.01(**), P≤0.001(***)

To validate this drug combination therapy strategy in *vivo*, BALB/c nude mice bearing MDA-MB-231 cell-derived tumors in the mammary fat pad and lung were randomly assigned into groups (*n* = 5 per group), receiving injection of PBS, cynaroside, amivantamab, and cynaroside+amivantamab respectively (Fig. 7A). Tumor tissues, major organs and plasma were collected after 14 days. Interval monitoring of tumor growth curves (Fig. 7B) and tumor imaging (Fig. 7C) revealed that while single-drug treatment with either cynaroside or amivantamab demonstrated tumor growth inhibition, the tumor inhibitory effect was significantly elevated after giving the two drugs in combination. At the same time, the number of metastatic tumors in the lungs of mice was significantly reduced after the combination of the two drugs (Fig. 7E-F). Next, co-IP and IHC staining were performed using mouse tumor tissue. Results showed that the binding capacity of EGFR and MET was significantly reduced in the cynaroside and cynaroside+amivantamab groups (Fig. 7G). IHC analysis of tumor sections showed the phosphorylation level of EGFR and MET and their downstream signaling components expression were significantly decreased after combination therapy, along with reduced Ki-67 proliferation index in both single-drug and combination groups (Fig. 7H-I). Importantly, no significant intergroup differences in body weight gain were observed throughout the treatment period (Fig.7E). Hematoxylin and eosin (H&E) staining further confirmed no apparent morphological damage in vital organs (heart, liver, spleen and kidneys) across all treatment groups (Fig. S4A). Biochemical blood indicators revealed that following combined treatment with the two drugs, AST levels in liver function markers and CK and LDH levels in cardiac function markers showed moderate elevation in mice, though all remained within normal reference ranges. The treatment had minimal impact on renal function, blood glucose, and blood lipid levels (Fig. S4B). In conclusion, the cynaroside-amivantamab combination effectively suppresses TNBC progression in mice models while maintaining a favorable safety profile.

## Discussion

EGFR, which exhibits a high positivity rate in TNBC, has been considered a promising therapeutic target [3, 25]. However, EGFR inhibitors have demonstrated limited clinical efficacy in TNBC. Crosstalk among RTKs and compensatory activation are thought to underlie this therapeutic resistance [26, 27]. In this study, we revealed that inhibition of the function of m6A-binding protein IGF2BP3 might be the key point to overcome TKI resistant in TNBC. It may restrict EGFR inhibitors sensitivity in TNBC through coordinating the expression of multiple RTKs in an m6A-dependent manner. So, we accordingly screened the small molecule compound cynaroside to block IGF2BP3-mRNA interactions. Moreover, by combining cynaroside with the EGFR/MET dual-target inhibitor amivantamab, we established a novel combinatorial therapeutic strategy for TNBC treatment.

IGF2BP3 is an m6A-binding protein specifically overexpressed in TNBC, where it promotes various biological processes including proliferation, metastasis, and stemness maintenance [19, 20, 28]. These effects mainly occur through IGF2BP3 binding to m6A sites on targeted RNAs, thereby regulating RNAs stability, splicing, and translation [21]. In our previous study, we found that IGF2BP3 could bind to the RNA m6A sites, cooperating with eIF4G2 to enhance MET mRNA translation initiation [23] and stabilizing FZD1/7 transcripts [29]. IGF2BP3 was also found to promote EGFR mRNA stability in an m6A-dependent manner by collaborating with METTL14 in colorectal cancer [30]. IGF2BP3 could also regulate IGF1R translation by binding IGF1R mRNA in Ewing sarcoma [31]. Although IGF2BP3 was found to modulate gene expression at different points across these studies, it consistently regulated multiple RTKs by binding specific sites on their mRNAs [32]. Based on our IGF2BP3 RIP/MeRIP-seq data, we identified that IGF2BP3 extensively regulated several RTKs such as EGFR, MET, and FGFR1 in an m6A-dependent manner in TNBC. This regulation altered cellular pan-tyrosine phosphorylation levels, providing a critical rationale for co-regulating RTKs to overcome EGFR resistance in TNBC.

In previous studies, JQ1, isoliquiritigenin, and d-ICD were identified as effective inhibitors of IGF2BP3 [33–35]. JQ1 suppress IGF2BP3 gene expression by targeting bromodomains of the BET family members, thereby inhibiting glioma progression [33]. Isoliquiritigenin reduces IGF2BP3 expression by suppressing N6-methylation levels, thereby controlling non-small cell lung cancer progression [34]. d-ICD inhibits drug resistance in hepatocellular carcinoma by downregulating IGF2BP3 expression [35]. Although these inhibitors can inhibit tumor progression by reducing IGF2BP3 expression, they do not directly target IGF2BP3 to block its RNA-binding activity. Their mechanisms are indirect and dependent on upstream molecular regulation. Previous studies indicated that the KH domains of IGF2BP3 were primarily responsible for its RNA binding and regulatory functions [22]. Each KH domain of IGF2BP3 comprises three α helices and three anti-parallel β-strands, which form a hydrophobic pocket for stable RNA attachment [36]. In this study, we also confirmed the binding of EGFR/MET mRNAs to the IGF2BP3 KH domains in TNBC. So, using the hydrophobic RNA-binding pocket within the KH1-2 domains as a docking site, we performed structure-based virtual screening to identify small-molecule inhibitors that competitively disrupt RNA-protein interactions, which provided a direct way to target and inhibit IGF2BP3 function in TNBC.

Through computerized virtual docking and experimental validation, cynaroside was identified as a novel small-molecule inhibitor that directly targeted the RNA binding function of IGF2BP3. Cynaroside, a flavonoid compound, is primarily used to treat chronic obstructive pulmonary disease, bronchial asthma, chronic cough associated with chronic pharyngitis and allergic rhinitis [37]. Its anti-tumor effects were also reported. For example, it induces G1-phase cell cycle arrest by downregulating CDC25A expression to inhibit colorectal cancer cell proliferation [38]; suppresses the MET/AKT/mTOR axis, thereby regulating gastric cancer cell proliferation, apoptosis, migration, and invasion [39]. However, these studies did not identify the direct molecular target of cynaroside. Here, we discovered its cytotoxic effects against TNBC and elucidated its precise mechanism of action by targeting IGF2BP3. Moreover, since members of the IGF2BP family share a conserved KH domain, the functional inhibitory effects of cynaroside could not limited to IGF2BP3 [40]. MST experiments confirmed that cynaroside exhibited high affinity for IGF2BP2 as well. Both IGF2BP2 and IGF2BP3 are proteins specifically overexpressed in TNBC, and significantly promote TNBC progression [41]. The functional inhibition of both proteins by cynaroside offers a more effective therapeutic approach for TNBC.

Although EGFR is highly expressed in TNBC, the specific overexpression of MET in TNBC remains equally noteworthy. Meta-analytical evidence indicates that MET overexpression is associated with a 1.29-fold increased risk of TNBC recurrence and a 1.38-fold increased risk of mortality [42]. MET was found to maintain phosphorylation activation of the EGFR downstream pathway through activation of ERBB3, thereby bypassing the blocking effect of EGFR inhibitors [43]. While cetuximab or panitumumab effectively inhibits EGFR activity through targeted therapy, its single-target mechanism fails to concurrently suppress MET functionality. Fortunately, the fourth-generation EGFR-targeting agent amivantamab was approved for marketing [44]. Amivantamab, an EGFR/MET bispecific antibody, simultaneously targets the extracellular ligand-binding domains of EGFR and MET, inhibiting their interactions with ligands and suppressing downstream signaling activation [45]. As a drug that can simultaneously target the two RTKs that are specifically and highly expressed in TNBC [11], amivantamab is undoubtedly a good candidate for the targeted treatment of TNBC. Moreover, based on this study, IGF2BP3 could co-regulate multiple RTKs and the core ones in TNBC were also EGFR and MET. The combined use of IGF2BP3 inhibitor cynaroside and amivantamab in TNBC not only dually suppressed EGFR/MET function by inhibiting expression levels and protein activity, but also reduced the risk of bypass resistance mediated by alternative pathways through cynaroside’s broad-spectrum inhibition of multiple RTKs. After experimental confirmation, the combination treatment with cynaroside and amivantamab exhibited highly synergistic effects, thereby establishing a rational combinatorial strategy for TNBC.

However, the drug regimens that can be translated into specific clinical applications require further investigation. First, in animal experiments, we administered amivantamab at 30 mg/kg every 4 days and cynaroside at 50 mg/kg every 2 days as both monotherapy and combination therapy. Although these doses were based on previous studies regarding drug treatment levels in mice [24, 39, 46, 47], they were both on the high level. While no significant impacts on body weight or major organ function after combined drug administration, liver and cardiac function showed elevated levels under this short-term dosing regimen. This suggests the need to identify a safer combination concentration for co-administration. Furthermore, in Lin et al.‘s study, intravenous cynaroside exhibited rapid distribution and slow decay in rat plasma, dissipating by 6 h post-administration. However, oral administration reached peak plasma concentration within 5 min, remained detectable for 12 h, and demonstrated about 26% oral bioavailability [48]. As an approved drug, amivantamab carries an FDA-recommended human dosage of 350 mg every two weeks for normal body weight and achieve steady-state concentrations after two months [44]. This suggests that oral cynaroside combined with less frequent amivantamab injections may offer a more suitable therapeutic approach.

**Fig. 8:**
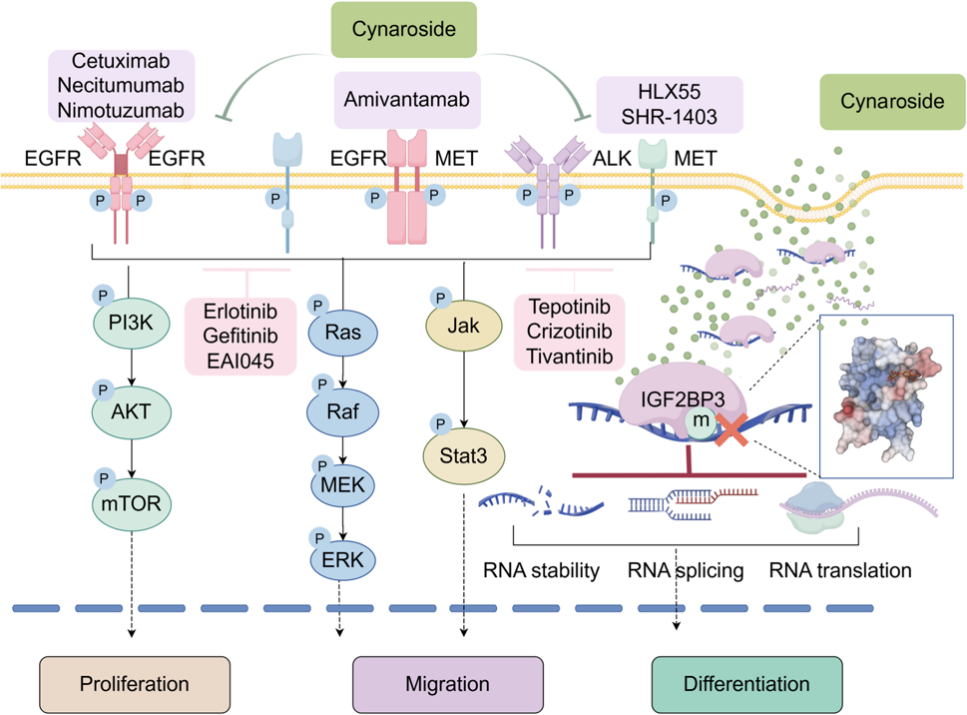
A proposed model of combining cynaroside with TKI to targeted therapy for TNBC. IGF2BP3 could coordinate the expression of multiple RTKs in an m6A-dependent manner and thus be the key point to address EGFR inhibitor resistance in TNBC. Cynaroside was screened to block IGF2BP3-mRNA interactions and inhibit RTK mRNA stability, splicing or translation. By combining cynaroside with TKIs, the phosphorylation level of PI3K, MAPK and Jak pathways were decreased and affected TNBC progression

Also, it is important to note that we have only proposed a feasible combination therapy based on the existing mechanisms. This does not imply that cynaroside combined with amivantamab is the sole treatment option under this mechanism. Novel inhibitors targeting EGFR, MET and other targets continue to emerge, and multiple pan-RTK inhibitors have been available (Fig. 8). We hope that our therapeutic strategy will facilitate broader screening for effective TKIs in TNBC, thereby expanding possibilities for targeted treatment approaches.

## Conclusion

In summary, our study identified IGF2BP3 as a key protein modulating resistance to EGFR inhibitors in TNBC and elucidated that IGF2BP3 induced this process through co-regulating multiple RTKs in m6A-dependent manner. Furthermore, we screened and validated the small-molecule inhibitor cynaroside as a targeted agent against IGF2BP3, which enhanced the sensitivity of TKIs in TNBC treatment by regulating the phosphorylation of multiple signaling pathways (Fig. 8). Additionally, by combining EGFR/MET bispecific antibody amivantamab with cynaroside, the core RTKs in TNBC were inhibited both in terms of expression level and protein activity, also, the risk of resistance due to bypass substitution was reduced by co-suppressing multiple RTKs. This established a rational combinatorial strategy for the targeted treatment of TNBC.

## Supplementary Information

Supplementary Material 1.

## Data Availability

No datasets were generated or analysed during the current study.

## Abbreviations

EGFR: Epidermal growth factor receptor
TNBC: Triple-negative breast cancer
RTK: Receptor tyrosine kinase
HNSCC: Neck squamous cell carcinoma
ER: Estrogen
PR: Progesterone
HER2: Human epidermal growth receptor-2
m6A: N6-methyladenosine
IGF2BPs: Insulin-like growth factor 2 mRNA-binding proteins
GEO: Gene Expression Omnibus
TKI: Tyrosine kinase inhibitor
AUC: Area under concentration-time curve
TCGA: The cancer genome atlas
PDB: Proteomic data commons
PBS: Phosphate buffered saline
H&E: Hematoxylin-eosin
IHC: Immunohistochemistry
GFP: Green fluorescent protein
cDNA: Complementary DNA
BCA: Bicinchoninic acid
HRP: Horseradish peroxidase
RIP: RNA immunoprecipitation
MeRIP: Methylated RNA immunoprecipitation
co-IP: Co-immunoprecipitation
CCK-8: Cell Counting Kit-8
CI: Combination index
IC50: Half maximal inhibitory concentration
DMSO: Dimethyl sulfoxide
MST: Microscale thermophoresis
KD: Dissociation constant
PDX: Patient-derived xenograft
