# Supplementary material for: Combinatorial inhibition of IGF2BP3-regulated receptor tyrosine kinases offers a new therapeutic strategy for triple-negative breast cancer
Source: J Exp Clin Cancer Res. 2026 Mar 11;45:99. doi: 10.1186/s13046-026-03686-7 (PMC13088498; doi:10.1186/s13046-026-03686-7)
Supplement: Supplementary file 1 — Supplementary Material 1. [file 13046_2026_3686_MOESM1_ESM.docx]

**Supporting Information**


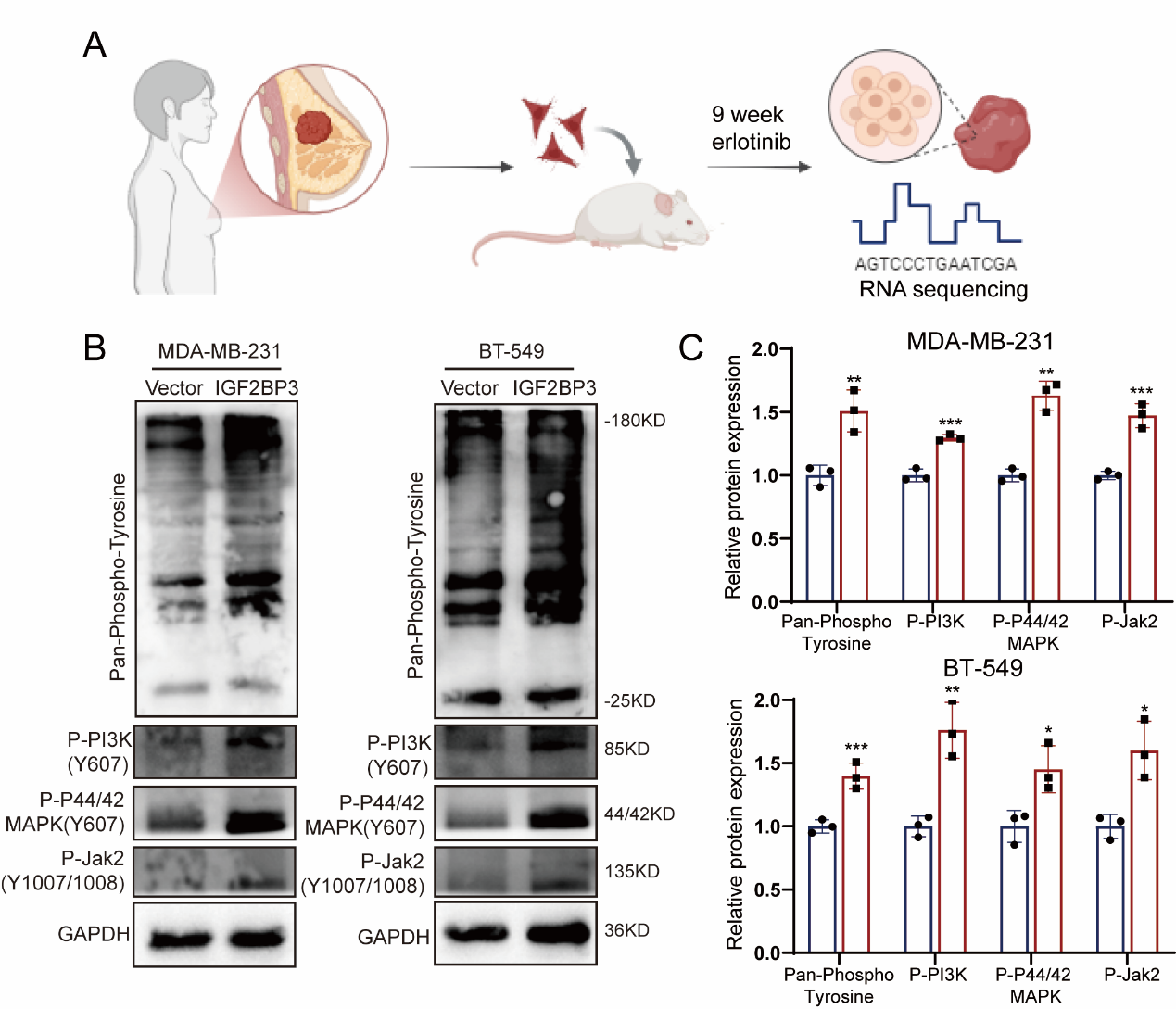


**Figure S1: IGF2BP3 affected pan-tyrosine phosphorylation.**

A. Modeling approach in GSE189257: human TNBC tissue xenografts were taken from mice and given 9 weeks erlotinib treatment followed by bulk RNA-seq. Image elements provided by biorender. B-C. Pan-tyrosine and PI3K/MAPK/Jak2 phosphorylation were increased after IGF2BP3 overexpression in MDA-MB-231 and BT-549 cell lines.


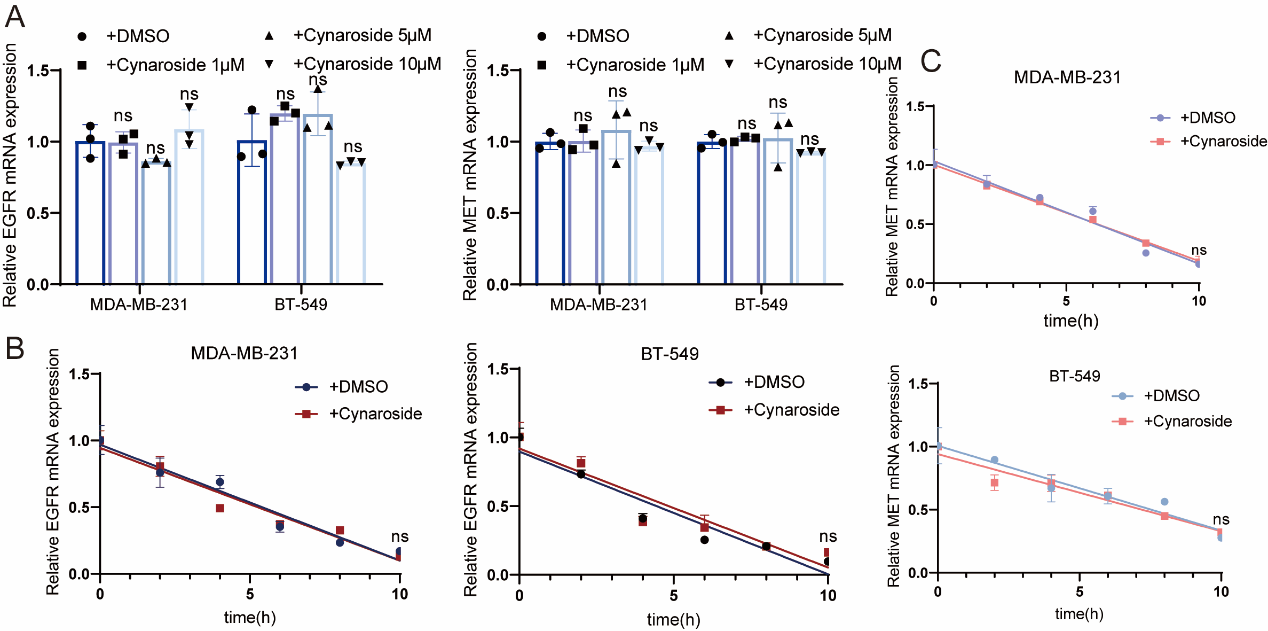


**Figure S2: Cynaroside had no effect on EGFR/MET RNA expression or stability.**

**A.** EGFR and MET mRNA levels was demonstrated by qRT-PCR after adding cynaroside to the gradient (0, 1, 5, 10 μM). B-C. MDA-MB-231 and BT549 cells which treated with 10μM cynaroside were added 5 μg/mL ActD for 0, 2, 4, 6, 8, 10 h, EGFR (B) and MET (C) mRNA stability was calculated by qRT-PCR.


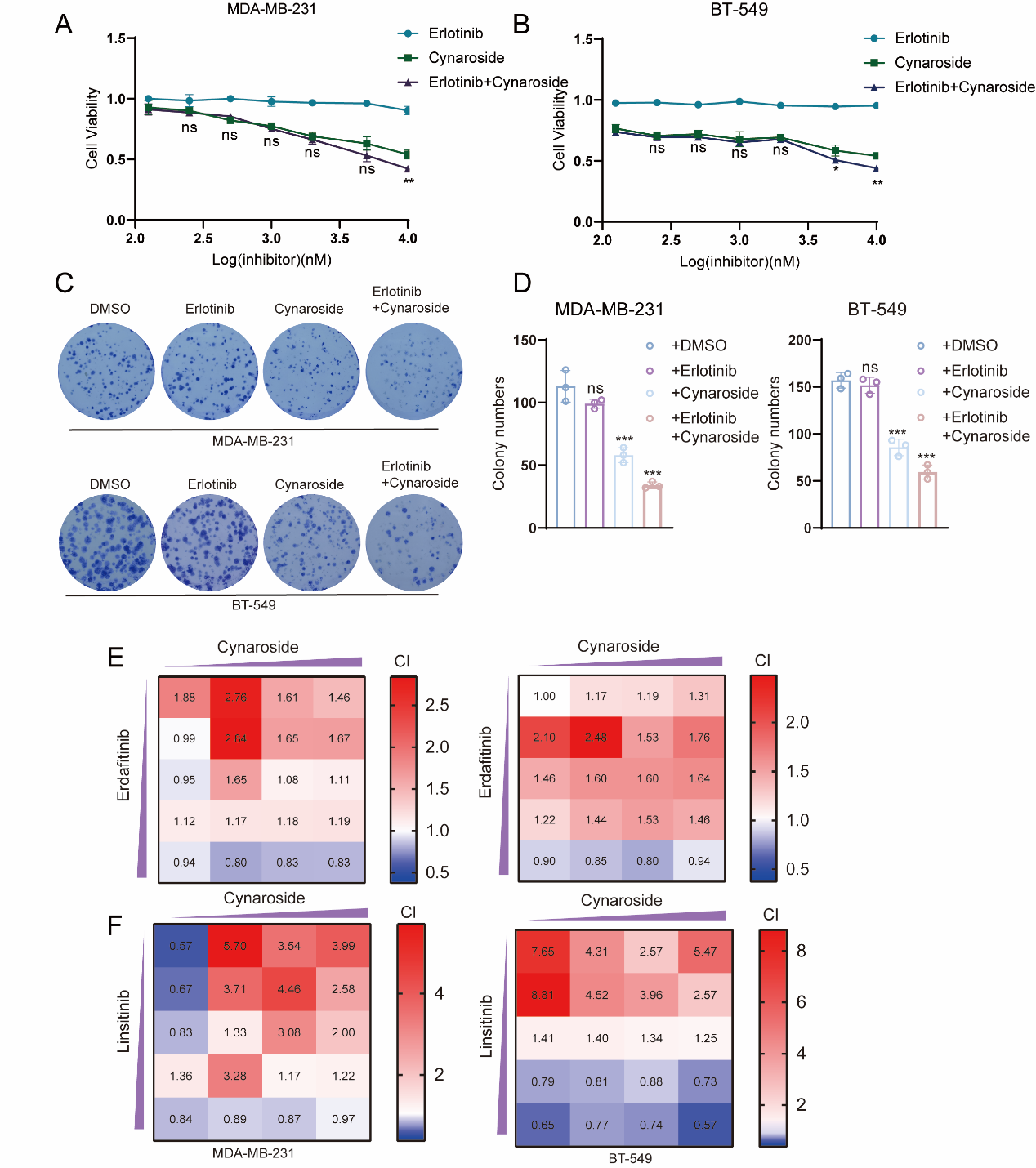


**Figure S3: Cynaroside effectively enhanced erlotinib efficacy in TNBC.**

A-B. MDA-MB-231 and BT-549 cell viabilities were detected after gradient addition of erlotinib, cynaroside and cynaroside+ erlotinib. C-D. Images (C) and quantifications (D) of cell colony numbers after erlotinib, cynaroside and cynaroside+ erlotinib treatment. Data were shown as mean ± SEM. E-F. Growth inhibition and CI of MDA-MB-231 and BT549 cells at different drug concentrations. Cynaroside concentrations were 3, 6, 12 and 15 μM from left to right. Erdafitinib (E) and Linsitinib (F) concentrations were 1.25, 2.5, 5, 10, 20 μM from top to bottom.


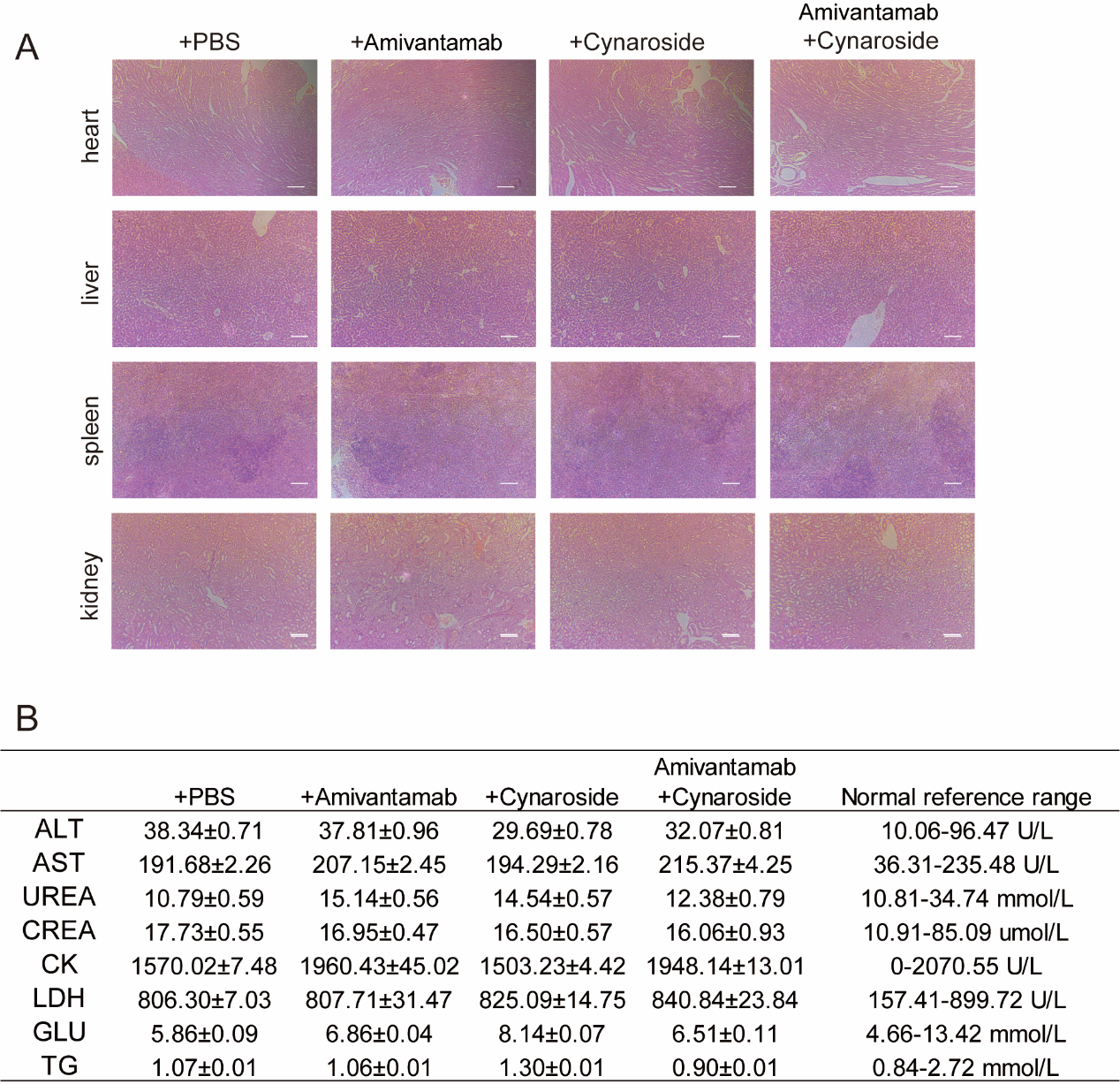


**Figure S4: Mouse exhibited stable vital signs under both single-agent and combination drug treatments.**

A. H&E staining further confirmed morphological damage in vital organs (heart, liver, spleen, and kidneys) across cynaroside, amivantamab and cynaroside+amivantamab groups (n=5). B. Plasma concentrations of ALT, AST, UREA, CREA, CK, LDH, GLU, and TG in different groups (n=5) of mice and their normal reference ranges. Values were expressed as mean ± SD.

**Table S1： Plasmids sequences**

| Name | Sequences (5’-3’) |
| --- | --- |
| ΔRRM1-2 | AAGCTTGccaccatgaacaaactgtatatcggaaacctcagcgagaacgccgccccctcggacctagaaagtatcttcaaggacgccaagatcccggtgtcgggacccttcctggtgaagactggctacgcgttcgtggactgcccggacgagagctgggccctcaaggccatcgaggcgctttcaggtaaaatagaactgcacgggaaacccatagaagttgagcactcggtcccaaaaaggcaaaggattcggaaacttcagatacgaaatatcccgcctcattta  cagtgggaggtgctggatagtttactagtccagtatggagtggtggagagctgtgagcaagtgaacactgactcggaaactgcagttgtaaatgtaacctattccagtaaggaccaagctagacaagcactagacaaactgaatggatttcagttagagaatttcaccttgaaagtagcctatatccctgatgaaatggccgcccagcaaaaccccttgcagcagccccgaggtcgccgggggcttgggcagaggggctcctcaaggcaggggtctccaggatccgtatccaagcagaaaccatgtgatttgcctctgGAATTC |
| ΔKH1-2 | AAGCTTGccaccATGctgcgcctgctggttcccacccaatttgttggagccatcataggaaaagaaggtgccaccattcggaacatcaccaaacagacccagtctaaaatcgatgtccaccgtaaagaaaatgcgggggctgctgagaagtcgattactatcctctctactcctgaaggcacctctgcggcttgtaagtctattctggagattatgcataaggaagctcaagatataaaattcacagaagagatccccttgaagattttagctcataataactttgttggacgtcttattggtaaagaaggaagaaatcttaaaaaaattgagcaagacacagacactaaaatcacgatatctccattgcaggaattgacgctgtataatccagaacgcactattacagttaaaggcaatgttgagacatgtgccaaagctgaggaggagatcatgGAATTC |
| ΔKH3-4 | AAGCTTGccaccatgaagaaaatcagggagtcttatgaaaatgatattgcttctatgaatcttcaagcacatttaattcctggattaaatctgaacgccttgggtctgttcccacccacttcagggatgccacctcccacctcagggcccccttcagccatgactcctccctacccgcagtttgagcaatcagaaacggagactgttcatctgtttatcccagctctatcagtcggtgccatcatcggcaagcagggccagcacatcaagcagctttctcgctttgctggagcttcaattaagattgctccagcAgaagcaccagatgctaaagtgaggatggtgattatcactggaccaccagaggctcagttcaaggctcagggaagaatttatggaaaaattaaagaagaaaactttgttagtcctaaagaagaggtgaaacttgaagctcatatcagagtgccatcctttgctgctggcagagttattggaaaaggaggcaaaacggtgaatgaacttcagaatttgtcaagtgcagaagttgttgtccctcgtgaccagacacctgatgagaatgaccaagtggttgtcaaaataactggtcacttctatgcttgccaggttgcccagagaaaaattcaggaaattctgactcaggtaaagcagcaccaacaacagaaggctctgcaaagtggaccacctcagtcaagacggaagGAATTC |

**Table S2: Antibody used in WB, IP and IHC**

| Name | manufacturer | **Cat No.** |
| --- | --- | --- |
| IGF2BP3 | proteintech | 14642-1-AP |
| GAPDH | proteintech | 60004-1-Ig |
| Pan-Phospho-Tyrosine | Abclonal | AP1162 |
| EGFR  P-EGFR | Proteintech  HuaBio | 18986-1-AP  ET1612-30 |
| MET  P-MET | Proteintech  Proteintech | 25869-1-AP  30737-1-AP |
| P-PI3K | Affinity | AF3242 |
| P-P44/42 MAPK | HuaBio | ET1610-13 |
| P-Jak2  IGF1R  INSR  PDGFRB  ROR1  FGFR1 | Abclonal  Proteintech  Proteintech  HuaBio  HuaBio  Proteintech | AP0373  66283-1-Ig  20433-1-AP  ET1605-20  HA601447  60325-1-Ig |
| Ki-67 | Servicebio | GB121141-100 |

**Table S3: The primer sequence of qRT-PCR**

| Name | Sense（5’-3’） |
| --- | --- |
| INSR | F: TCTTGGACGGAACCCACCTA  R: CCCACCATGCTCAGTGCTAA |
| MET | F: CAGTCGGAGGTTCACTGCAT  R: CTGGATGGGAGTCCAGGAGA |
| EGFR | F: aacgaatgggcctaagatcc  R: actgggtgtaagaggctcca |
| β-actin | F: TCACCCACACTGTGCCCATCTACGA  R:CAGCGGAACCGCTCATTGCCAATGG |
| PDGFRB | F: AGCAGGTCAGAACGAAGGTG  R: AGCCCCTGTTTCCTGATGTC |
| ROR1 | F: CCTTTCGGATGCAAAGCTGTC  R: GGTAGCTCTTTAGCCTTGCTCT |
| FGFR1 | F: ggcctctatgcttgcgtaac  R: gagctacgggcatacggttt |
| IGF1R | F: CACAGGCACACAGGTCTCAT  R: CCCATGGCCACCAGATTCTT |
